# Dr. Josiah Ramsey Is Dead

**Published:** 1899-11

**Authors:** 


### BIOGRAPHICAL.

### Dr. Josiah Ramsey is Dead.

DEMISE OF ONE OF SPRINGFIELD'S MOST RESPECTED PIONEER CITIZENS.

Dr. Josiah Ramsey, the oldest dentist in the city, died of pneumonia, Wednesday, September 27th, at 10 o'clock P. M., at his residence, 920 East High Street, after a brief illness.

Dr. Ramsey was born near Harrisburg, Pa., May 12, 1816.

He came to Ohio in 1836. For several years he taught school near Dayton, Ohio, also near Natchez, Miss. Returning to Cincinnati, he went into the office of Drs. Edward and James Taylor. He soon after completed a course in the Ohio Dental College, and began the practice of dentistry in this city in August, 1850.

By his skill and good judgment he soon earned for himself the highest eminence in his profession, and was known as one of the best dentists of Ohio. He practiced dentistry in this city for forty-nine years, occupying the same rooms in the Fisher Building for twenty-six years. On account of failing health, in August, 1893, he moved his office to his residence on East High Street, where he continued his practice until the time of his death.

He was a member of the State Dental Association, and while he has not been able to attend the meetings for the last few years, yet he enjoyed reading about them. He was a strong Republican, never missing to cast his vote, unless he was on a sick bed and not able to be moved.

He was also a charter member of the Second Presbyterian Church, and was a useful and faithful member. His life was like the quiet shining of a star. None can tell how many were blessed and helped by the quiet, steady influence of his Christian life.

He was married to Mrs. Emma M. Steele, at Pine Grove Furnace, near Hanging Rock, Ohio, September 23, 1857, who, with his two daughters, Miss M. Jennie B. Ramsey and Mrs. Jas. M. Rumsey, of Rawlins, Wyoming, are left to mourn his loss.

The funeral took place Friday afternoon at 2 o'clock from his late residence, 920 East High Street. The services were performed by the Rev. Edwin P. Thomson, pastor of the Second Presbyterian Church, also Rev. Dr. Geo. Fullerton, of the Third Presbyterian Church, after which the funeral train proceeded to Ferncliff Cemetery, where the body was laid to rest with solemn and impressive ceremonies.

In addition to the above statement it may be said that, though Dr. Ramsey was quiet in demeanor, never very demonstrative, yet was sincere and earnest in whatever he did or undertook ; he possessed a genial, social disposition.

In his intercourse with his professional brethren he was rather retiring, never aggressive, yet whenever occasion arose, he was ready to give his opinion and the reason for it, and generally his views and judgment were accepted without criticism or controversy.

It was the good fortune of the writer of these lines to be a class mate in the Ohio Dental College with Dr. Ramsey, and he was the last but one of the class now living.

The days of our college life, having such members as he, have left memories that will never be blotted out.

He leaves an example of true manhood and Christian character that may well be imitated by all. Such as he, in their influence, do not die at their passage from this life, but still speak to those left behind. J. T.

---

### DR. JOSIAH RAMSEY.

---

### RESOLUTIONS ADOPTED BY THE SPRINGFIELD DENTAL ASSOCIATION IN MEMORY OF THE LATE DR. RAMSEY.

---

At a meeting of the dentists of Springfield, O., September 28, 1899, the following resolutions on the death of Dr. Josiah Ramsey were adopted :

*Resolved*, That in his death the profession has lost one of its oldest, most able, respected members, being in continuous practice of his profession for nearly half a century.

*Resolved*, That we extend to his bereaved family our sincere sympathy in their hour of affliction.

*Resolved*, That a copy of these resolutions be sent to the family of the deceased, to the city papers, and also to the dental journals for publication.

